# Site preference and tetragonal distortion in palladium-rich Heusler alloys

**DOI:** 10.1107/S2052252518017578

**Published:** 2019-01-24

**Authors:** Mengxin Wu, Yilin Han, A. Bouhemadou, Zhenxiang Cheng, R. Khenata, Minquan Kuang, Xiangjian Wang, Tie Yang, Hongkuan Yuan, Xiaotian Wang

**Affiliations:** a School of Physical Science and Technology, Southwest University, Chongqing 400715, People’s Republic of China; b Laboratory for Developing New Materials and Their Characterization, University Ferhat Abbas Setif 1, Setif 19000, Algeria; c Institute for Superconducting and Electronic Materials (ISEM), University of Wollongong, Wollongong 2500, Australia; d Laboratoire de Physique Quantique de la Matière et de Modélisation Mathématique (LPQ3M), Université de Mascara, Mascara 29000, Algeria; e Applied Physics, Division of Materials Science, Department of Engineering Sciences and Mathematics, Luleå University of Technology, Luleå SE-971 87, Sweden

**Keywords:** full-Heusler alloys, L2_1_ structures, XA structures, tetragonal distortion, computational modeling, inorganic materials, density functional theory, structure prediction

## Abstract

In this work, two kinds of competition between different Heusler structure types are considered, one is the competition between XA and L2_1_ structures based on the cubic system of full-Heusler alloys, Pd_2_ *YZ* (*Y* = Co, Fe, Mn; *Z* = B, Al, Ga, In, Tl, Si, Ge, Sn, Pb, P, As, Sb). Most alloys prefer the L2_1_ structure; that is, Pd atoms tend to occupy the *a* (0, 0, 0) and *c* (0.5, 0.5, 0.5) Wyckoff sites, the *Y* atom is generally located at site *b* (0.25, 0.25, 0.25), and the main group element *Z* has a preference for site *d* (0.75, 0.75, 0.75), meeting the well known site-preference rule. The difference between these two cubic structures in terms of their magnetic and electronic properties is illustrated further by their phonon dispersion and density-of-states curves. The second type of competition that was subjected to systematic study was the competitive mechanism between the L2_1_ cubic system and its L1_0_ tetragonal system. A series of potential tetragonal distortions in cubic full-Heusler alloys (Pd_2_ *YZ*) have been predicted in this work. The valley-and-peak structure at, or in the vicinity of, the Fermi level in both spin channels is mainly attributed to the tetragonal ground states according to the density-of-states analysis. Δ*E*_M_ is defined as the difference between the most stable energy values of the cubic and tetragonal states; the larger the value, the easier the occurrence of tetragonal distortion, and the corresponding tetragonal structure is stable. Compared with the Δ*E*_M_ values of classic Mn_2_-based tetragonal Heusler alloys, the Δ*E*_M_ values of most Pd_2_Co*Z* alloys in this study indicate that they can overcome the energy barriers between cubic and tetragonal states, and possess possible tetragonal transformations. The uniform strain has also been taken into consideration to further investigate the tetragonal distortion of these alloys in detail. This work aims to provide guidance for researchers to further explore and study new magnetic functional tetragonal materials among the full-Heusler alloys.

## Abstract 2

Two kinds of competition between different Heusler structure types are explored. The first is the competition between XA and L2_1_ structures based on the cubic system of full-Heusler alloys of Pd_2_ *YZ* and the second is the competitive mechanism between the L2_1_ cubic system and its L1_0_ tetragonal system.

## 1. Introduction   

Since the first series of Heusler compounds, of the general formula Cu_2_Mn*X* (*X* = Al, In, Sn, Sb, Bi), was proposed by Heusler in 1903, the passion for research in Heusler alloys has continued to rise because of their numerous excellent properties and potential for many applications in numerous technical fields. They act as promising candidates for spin-gapless semiconductors (Wang, Chang, Liu *et al.*, 2017 ▸; Bainsla *et al.*, 2015 ▸; Wang *et al.*, 2016 ▸; Gao & Yao, 2013 ▸; Skaftouros *et al.*, 2013*a* ▸), thermoelectric materials (Wehmeyer *et al.*, 2017 ▸; Lue *et al.*, 2007 ▸; Lue & Kuo, 2002 ▸), shape memory alloys (Li *et al.*, 2018 ▸; Aksoy *et al.*, 2009 ▸), superconductors (Nakajima *et al.*, 2015 ▸; Sprungmann *et al.*, 2010 ▸; Shigeta *et al.*, 2018 ▸) and topological insulators (Hou *et al.*, 2015 ▸; Lin *et al.*, 2015 ▸). Therefore, ongoing investigations of Heusler alloys are quite active and are set to continue due to predictions of their enhanced performance through theoretical design and experimental synthesis. Heusler alloys normally have three types of structure, full-Heusler, half-Heusler and quaternary Heusler, with stoichiometric compositions of *X*_2_ *YZ*, *XYZ* and *XYMZ*, respectively. Usually, the *X*, *Y* and *M* atoms are transition elements and the *Z* atom is a main group element.

As a classic type of Heusler alloy, full-Heusler alloys have been attracting much interest from researchers; however, the innovative properties of full-Heusler alloys depend strongly on their highly ordered structure. So, ignoring other factors, we now only consider this highly ordered structure to yield two possible atomic ordering figurations: the XA type [or the Hg_2_CuTi/inverse type, with space group 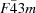 (No. 216)] and the L2_1_ type [or Cu_2_MnAl type, with space group 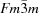 (No. 225)], represented stoichiometrically by *XXYZ* and *XYXZ*, respectively. According to the general site preference rule, for full-Heusler alloys represented by *X*_2_ *YZ*, if the valence electrons of *X* are more numerous than those of *Y*, *X* tends to occupy Wyckoff sites *a* (0, 0, 0) and *c* (0.5, 0.5, 0.5), but prefers sites *a* (0, 0, 0) and *b* (0.25, 0.25, 0.25) when *Y* possesses more valence electrons than *X*. However, some counter-examples were found: when *X* represents a low-valence metal in particular, *X* tends to occupy the *a* and *c* positions, forming an L2_1_ structure such as in Ti_2_Cr*Z*, Ti_2_Cu*Z* and Ti_2_Zn*Z* (Wang, Cheng, Yuan & Khenata, 2017 ▸). However, we found that the cubic competitive mechanism of XA and L2_1_ for *X*_2_ *YZ* alloys mostly focuses on the *X* elements with fewer valence electrons such as Ti and Sc. The occupation of the atomic position has been confirmed to have a great influence on the properties of the Heusler alloy (Qin *et al.*, 2017 ▸), so it is necessary to study the positioning in the *X*_2_ *YZ* Heusler alloys for *X* with numerous valence electrons such as in Cu, Ni and Pd.

On the other hand, a review of recent studies of Heusler compounds suggests that researchers are more interested in cubic structures than tetragonal structures; the progress made in finding better tetragonal phases is limited. However, the tetragonal phases have some excellent properties such as large magneto-crystalline anisotropy (Salazar *et al.*, 2018 ▸; Matsushita *et al.*, 2017 ▸), large intrinsic exchange-bias behavior (Felser *et al.*, 2013 ▸; Nayak *et al.*, 2012 ▸), high Curie temperature and low magnetic moment. In addition, it is reported that the tetragonal Heusler alloys have a large perpendicular magnetic anisotropy, which is the key to spin-transfer torque devices (Balke *et al.*, 2007 ▸). Therefore, searching for new, better tetragonal phases and studying their possible tetragonal transformations from the cubic phase are essential and fundamental.

In this work, a series of full-Heusler alloys, Pd_2_ *YZ* (*Y* = Co, Fe, Mn; *Z* = B, Al, Ga, In, Tl, Si, Ge, Sn, Pb, P, As, Sb), were chosen in order to study their atomic ordering competition between two cubic-type structures: the XA type and the L2_1_ type. Furthermore, the tetragonal transformation and phase stability of the above-mentioned alloys were also investigated by means of first principles. The origin of the tetragonal ground state of Pd_2_ *YZ* alloys is explained with the help of density of states (DOS).

## 2. Computational methods   

First-principle band calculations were carried out via the plane-wave pseudo-potential method (Troullier & Martins, 1991 ▸) using *CASTEP* code in the framework of density functional theory (Becke, 1993 ▸). The Perdew–Burke–Ernzerhof functional of the generalized gradient approximation (Perdew *et al.*, 1996 ▸) and ultra-soft (Al-Douri *et al.*, 2008 ▸) pseudo-potential were used to describe the interaction between electron-exchange-related energy and the nucleus and valence electrons, respectively. The integration over the first Brillouin zone was performed with a *k*-mesh grid of 12 × 12 × 12 for the cubic structure and 12 × 12 × 15 for the tetragonal structure, using a Monkhorst–Pack grid with a cut-off energy of 450 eV and a self-consistent field tolerance of 10^−6^ eV. The spin-polarization was also considered in the total energy calculation.

For the calculations of the phonon spectrum of Pd_2_-based Heusler alloys, we used the finite displacement method implemented in the *CASTEP* code. During the phonon spectrum calculations, the *k*-mesh grids of 12 × 12 × 12 and 12 × 12 × 15 in the Brillouin zone integration are used for cubic and tetragonal Heusler alloys, respectively.

## 3. Results and discussion   

### 3.1. The site ordering competition between XA and L2_1_ structures in the cubic phases of Pd_2_ *YZ* full-Heusler alloys   

**Figure 1:**
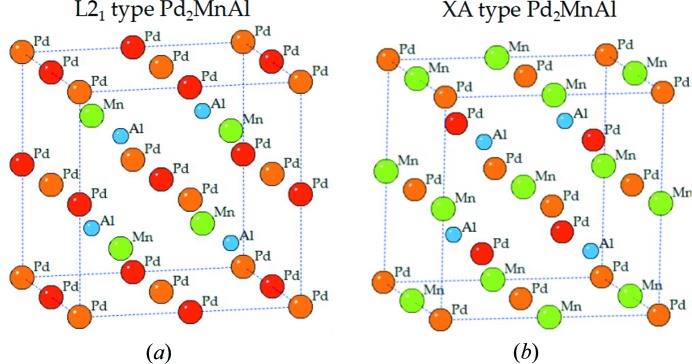
Crystal structures of L2_1_- and XA-type full-Heusler alloys of Pd_2_MnAl.

**Figure 2:**
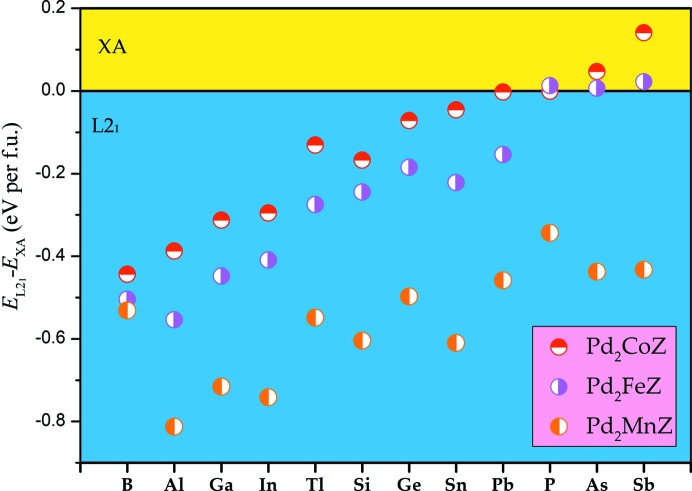
The difference in total energy for L2_1_ and XA types in Pd_2_-based alloys.

Since not all the full-Heusler alloys obey the site preference rule (Zhang *et al.*, 2016 ▸; Lukashev *et al.*, 2016 ▸; Meng *et al.*, 2017 ▸; Wang, Cheng & Wang, 2017 ▸), clarifying the preferable atomic ordering of these alloys is necessary. Two structural configurations of Pd_2_MnAl are given as examples in Fig. 1 ▸. The first is the L2_1_ structure, where Pd atoms carrying more valence electrons than Mn and Al atoms occupy Wyckoff sites *a* (0, 0, 0) and *c* (0.5, 0.5, 0.5), while the Mn atom is at site *b* (0.25, 0.25, 0.25) and the Al atom is located at site *d* (0.75, 0.75, 0.75); this meets the well known site-preference rule (Bagot *et al.*, 2017 ▸; Burch *et al.*, 1974 ▸). The second is the XA type, where Pd elements are at Wyckoff sites *a* and *b*. To clarify which is the favorable atomic ordering of Pd_2_ *YZ*, we calculated and plotted *E*_L2_1__ − *E*_XA_ as a function of different alloys in Fig. 2 ▸. When the value of the difference is negative, *E*_XA_ is larger than *E*_L2_1__, indicating that the L2_1_ phase is more stable than the XA phase due to the lower total energy. The inverse is also true. We can clearly see that most of these alloys prefer the L2_1_ phase, except for Pd_2_Co*Z* (*Z* = As, Sb, P, Pb) and Pd_2_Fe*Z* (*Z* = As, Sb, P) from Fig. 2 ▸. The positive difference values in Pd_2_CoAs, Pd_2_CoSb and Pd_2_FeSb imply that the XA state with lower energy is the most stable phase for the three alloys. We note that the difference values for Pd_2_CoP, Pd_2_CoPb, Pd_2_FeP and Pd_2_FeAs are around zero, which shows that these alloys have no obvious preferred steady state, and it is likely to be the state where the XA type and the L2_1_ type coexist. Moreover, the larger the absolute values of the difference, the more stable the steady state of the corresponding substance. In addition, as the atomic radius of *Y* atoms increases from Co to Fe to Mn, the absolute value of the difference mostly becomes larger, indicating that the stability of these alloys is enhanced.

**Figure 3:**
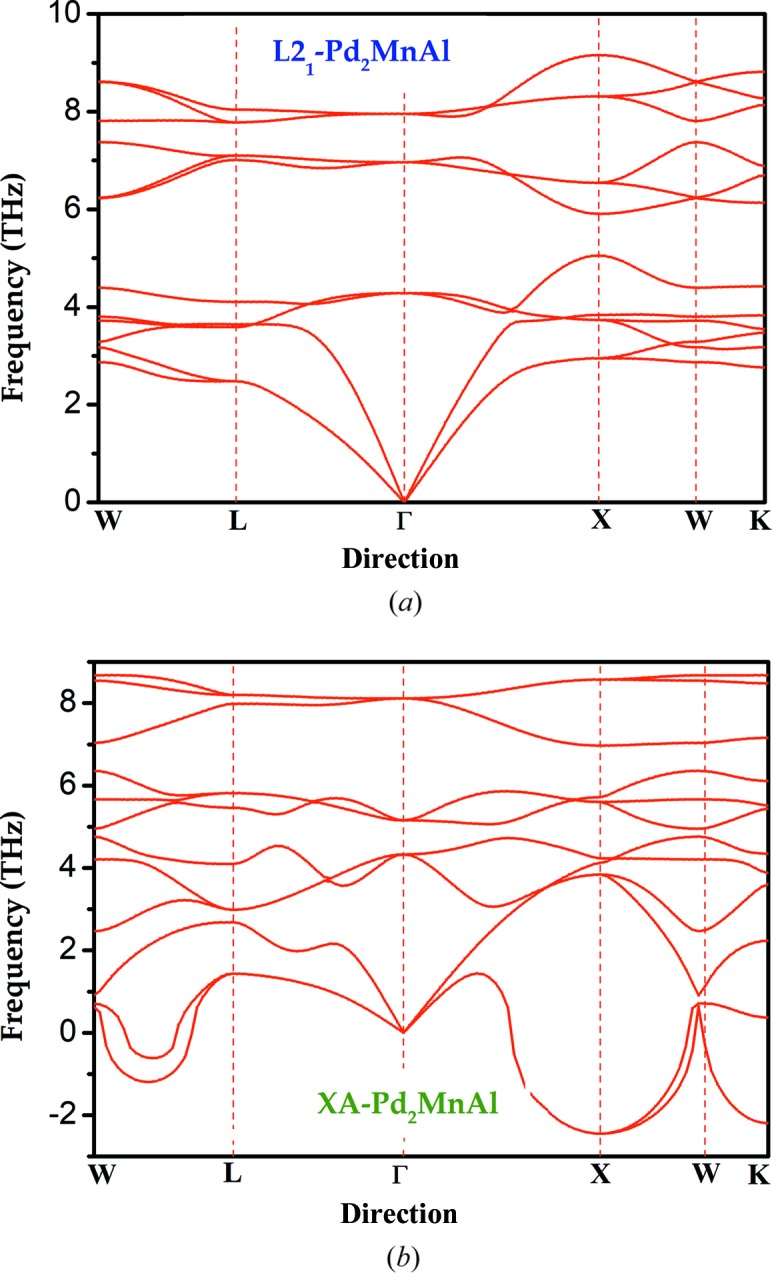
Calculated phonon dispersion curves for (*a*) L2_1_-Pd_2_MnAl and (*b*) XA-Pd_2_MnAl.

In order to further elucidate the dynamic stability of the alloys of interest, as an example we calculated the phonon dispersion curves of Pd_2_MnAl along the W-L-Γ-X-W-K directions for L2_1_- and XA-type structures in the Brillouin zone (displayed in Fig. 3 ▸). There are four atoms in a primitive cell of Pd_2_MnAl, resulting in 3 × 4 = 12 branches in its phonon dispersion curves, and each branch corresponds to a mode of vibration. Among these, the three low-frequency branches correspond to acoustic phonon curves, while the other nine high-frequency branches correspond to optical phonon curves. From Fig. 3 ▸ we can see that the phonon dispersion spectra for the L2_1_-Pd_2_MnAl compound have no imaginary frequencies, whereas Pd_2_MnAl in the XA-type structure has an imaginary frequency, which further proves that Pd_2_MnAl is stable in L2_1_ and unstable in the XA phase.

**Figure 4:**
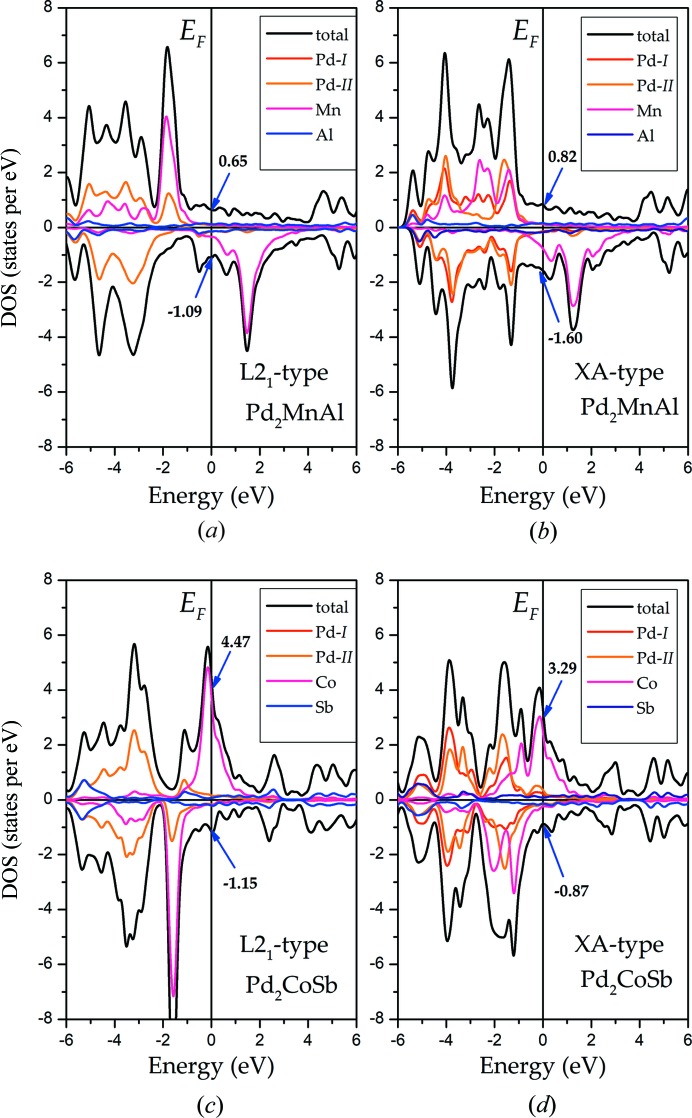
The total and atomic density of states (DOSs) of Pd_2_MnAl and Pd_2_CoSb in L2_1_ and XA structures, respectively.

In order to further explain the favorability of the atomic ordering, the DOS curves are given in Fig. 4 ▸. Pd_2_MnAl and Pd_2_CoSb were selected as examples; we found that whether in XA or L2_1_ type, the total magnetic moment arises mainly from the *Y* element – here, Mn and Co atoms – due to their strong exchange splitting (Zhao *et al.*, 2017 ▸) in the vicinity of *E*_F_. The magnetic moments of Al and Sb atoms are quite small, so they can be ignored. The almost-symmetry of the PDOSs of Pd in the spin-down channel makes Pd have a very small magnetic moment, so it also makes very little contribution to the total magnetic moment. Note that in the L2_1_-type structures, there is only one line of the two Pd atoms’ magnetic moments. In the L2_1_ states, two Pd atoms occupy sites *a* (0, 0, 0) and *c* (0.5, 0.5, 0.5); thus, the surrounding environments of the Pd atoms are the same based on the symmetry and periodicity of the structures, causing the two lines to be recombined into one. This situation does not exist in the XA structures. The valence electrons at, or in the vicinity of, the Fermi level mostly determine the magnetic and electronic structures of these full-Heusler alloys. In Figs. 4 ▸(*a*) and 4(*b*), the L2_1_-type structure of Pd_2_MnAl has lower energy in both the majority and minority spin channels than the XA type at *E*_F_, with 0.17 and 0.51 states per eV, respectively, indicating that Pd_2_MnAl is more stable in the L2_1_ type than in the XA type. Meanwhile, the situation is different in Pd_2_CoSb, with the value of DOS at or around *E*_F_ in the XA-type structure, being less than that in the L2_1_ type (1.18 and 0.28 states per eV) separately in the spin-up and spin-down channels, respectively – making it more stable in XA structures. These two alloys correspondingly exhibit L2_1_- and XA-type structures, respectively. Through calculation, we found that Pd_2_ *YZ* alloys mostly exhibit L2_1_-type structures.

### 3.2. Magnetic and Slater–Pauling rules of cubic-type Pd_2_ *YZ*   

**Figure 5:**
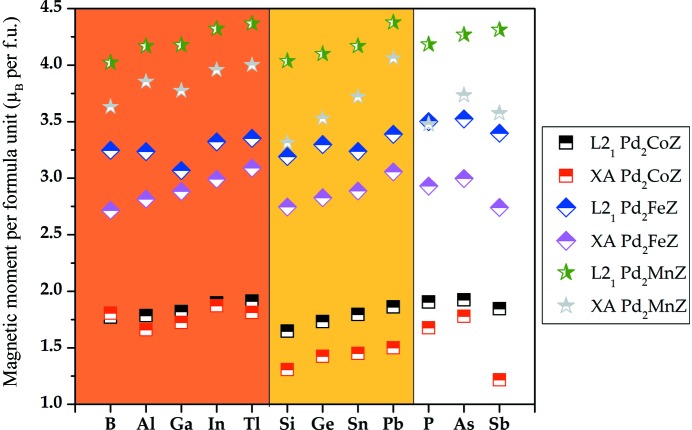
The total magnetic moment per formula unit as a function of the different Pd_2_-based alloys in both L2_1_ and XA structures.

To investigate the magnetic properties of Pd_2_ *YZ*, we plotted the total magnetic moment per formula unit as a function of different alloys in two cubic phases, the XA and L2_1_ states (Fig. 5 ▸). It is clear that all magnetic moments in the L2_1_ phases are larger than those in the XA type in certain alloys. Secondly, the magnetic moments of Pd_2_Mn*Z* (*Z* = B, Al, Ga, In, Tl, Si, Ge, Sn, Pb, P, As, Sb) – about 4 µ_B_ – are the largest, followed by Pd_2_Fe*Z* (*Z* = B, Al, Ga, In, Tl, Si, Ge, Sn, Pb, P, As, Sb), while Pd_2_Co*Z* (*Z* = B, Al, Ga, In, Tl, Si, Ge, Sn, Pb, P, As, Sb) alloys have the smallest magnetic moments at around 1.5 µ_B_. We also computed the total and atomic magnetic moments of the equilibrium lattice constants in the XA- and L2_1_-type structures of Pd_2_ *YZ*; these are listed in Tables S1 and S2 of the supporting information and show that the total magnetic moments mainly come from the *Y* elements owing to their large strong exchange splitting.

Moreover, the sums of the valence electrons of the two Pd and *Y* atoms are already 27, 28 and 29, corresponding to the three types of alloy, Pd_2_Mn*Z*, Pd_2_Fe*Z* and Pd_2_Co*Z*. So, if these alloys meet the famous Slater–Pauling rule (Galanakis *et al.*, 2014 ▸; Faleev *et al.*, 2017*a* ▸; Skaftouros *et al.*, 2013*b* ▸), they should obey the rule of *M*_t_ = *Z*_t_ − 28. These alloys should have a larger magnetic moment than current magnetic moments according to the Slater–Pauling rule, which suggests that this rule does not apply for Pd_2_ *YZ*. Furthermore, we should point out that all the Pd_2_ *YZ* alloys in this study are not half-metallic materials or spin-gapless semiconductors, and even the majority of Pd_2_-based alloys do not have half-metallic or spin-gapless semiconducting behaviors. The well known Slater–Pauling rule is a method of predicting half-metallic or spin-gapless semiconductor materials, and thus the Pd_2_ *YZ* (or even Pd_2_-based Heusler) alloys do not obey the Slater–Pauling rule.

### 3.3. Possible tetragonal transformations in Pd_2_ *YZ* compounds   

Stable tetragonal phases and possible tetragonal transformations are important for investigating Heusler alloys. Thus, we now discuss the possible tetragonal transformations in Pd_2_ *YZ*. Because most of these alloys are L2_1_-type stable, we applied tetragonal deformation and uniform strain to search for the tetragonal phases and possible tetragonal transformations in only L2_1_-type structures. We should point out here, for Pd_2_CoSb, Pd_2_CoAs and Pd_2_FeSb, the XA structure is much more stable than L2_1_; we also studied the possible tetragonal transformations in XA-type structures of these three alloys (see Fig. S1 of the supporting information).

**Figure 6:**
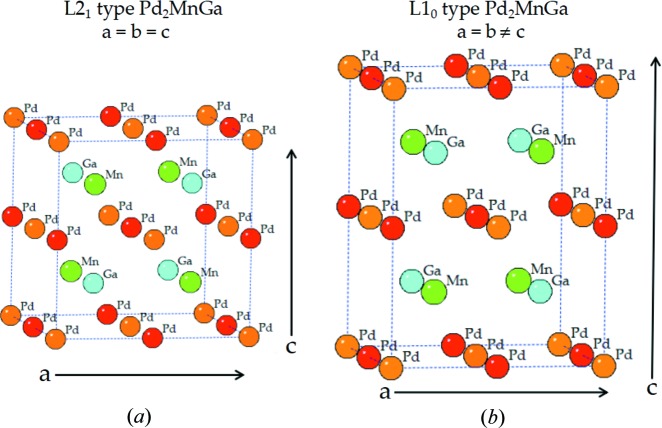
Crystal structures of (*a*) cubic L21 and (*b*) tetragonal L1_0_ Pd_2_MnGa.

**Figure 7:**
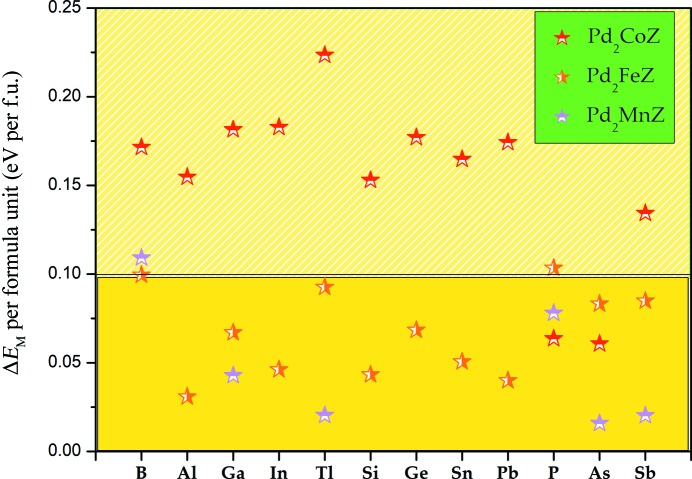
*E*_M_ per formula unit as a function of the different Pd_2_-based alloys.

By maintaining the volume of the tetragonal unit cell *V*_tetragonal_ = *a* × *b* × *c* (*a* = *b*) as equal to the equilibrium cubic volume *V*_equilibrium_ = *a* ^3^ while changing the *c*/*a* ratio, we obtain the L1_0_-type structures as shown in Fig. 6 ▸. We assume that the volume for the equilibrium state does not change with tetragonal distortions. During the tetragonal deformation, there are two important parameters: Δ*E*_M_ and the *c*/*a* ratio. Δ*E*_M_ is the difference in energy between the most stable cubic state and the most stable tetragonal phase; the total energy is set to zero at a *c*/*a* ratio of 1, which represents the most stable L2_1_-type cubic phase. By relaxing the *c*/*a* ratios, the minimum of the total energy can be obtained in the tetragonal distortion, which corresponds to the most stable tetragonal phase. It can be seen from Fig. 7 ▸ that almost all of the Pd_2_ *YZ* alloys [except for Pd_2_Mn(Al/In/Si/Ge/Sn/Pb)] can undergo tetragonal deformation and form a tetragonal Heusler L1_0_ structure.

**Figure 8:**
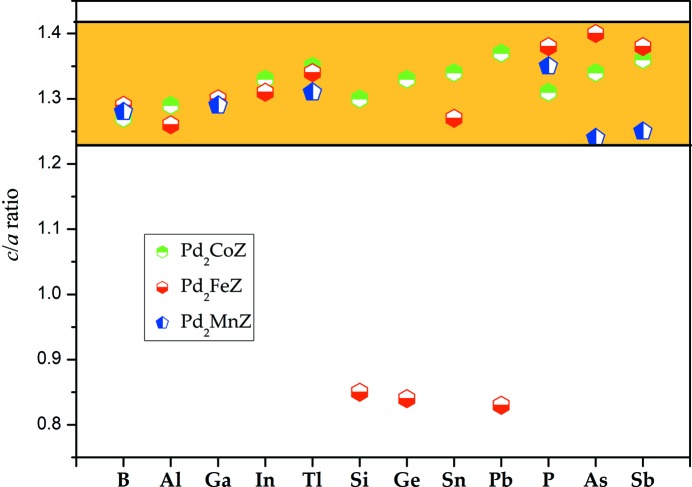
The *c*/*a* ratio as a function of the different Pd_2_-based alloys.

According to the classic tetragonal Heusler alloys, for the occurrence of a stable tetragonal phase, a relatively large Δ*E*_M_ is needed. Generally, an absolute value of Δ*E*_M_ ≥ 0.1 eV per formula unit (f.u.) is required for Mn_2_-based Heusler alloys; for example, the absolute values of Δ*E*_M_ for Mn_3_Ga (Liu *et al.*, 2018 ▸) and Mn_2_FeGa (Faleev *et al.*, 2017*b* ▸) are about 0.14 and 0.12 eV per f.u., respectively. We were excited to find that the vast majority of absolute values of Δ*E*_M_ for Pd_2_Co*Z* alloys are larger than 0.1 per f.u., which hints that for most Pd_2_Co*Z* alloys, we may not observe a cubic state for them in the experiment. We note that the maximum value of Δ*E*_M_ in these alloys appears in Pd_2_CoTi and is about 0.225 eV per f.u., almost two times that of Mn_2_FeGa. We also found that the stable tetragonal structures of Mn_3_Ga (Liu *et al.*, 2018 ▸), Mn_2_FeGa (Faleev *et al.*, 2017*b* ▸) and Zn_2_RuMn (Han *et al.*, 2019 ▸) occur at *c*/*a* = 1.30, 1.40 and 1.41, respectively; this indicates that the stable tetragonal phases of these Pd-based alloys occur in the reasonable *c*/*a* range of from 1.23 to 1.42, as shown in Fig. 8 ▸. However, there are also cases where the tetragonal transformation occurs at *c*/*a* < 1, such as for Pd_2_Fe*Z* (*Z* = Si, Ge, Pb). The curves of the tetragonal deformation of each alloy can be seen in Figs. S2, S3 and S4.

**Figure 9:**
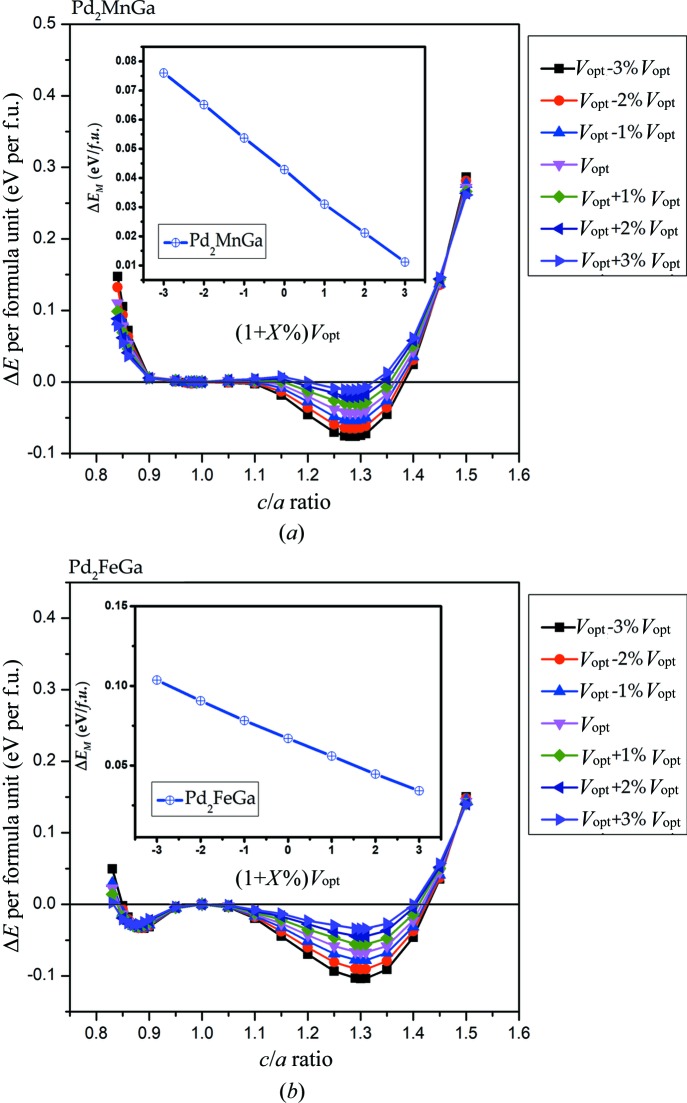
Total energies as functions of the *c*/*a* ratio for Pd_2_MnGa and Pd_2_FeGa with contraction/expansion of the unit-cell volume. The zero point of the total energy was set to that of the most stable L2_1_ cubic phase (*c*/*a* = 1).

Uniform strain has also been taken into consideration to study the possible tetragonal transformations. To facilitate the study of all the alloys, we take Pd_2_MnGa and Pd_2_FeGa as examples. Change in volume can influence the value of Δ*E*_M_ as shown in the inset of Fig. 9 ▸. Δ*E*_M_ and *V*_opt_ + *X*%*V*_opt_ are negatively correlated; that is, when *X* changes from −3 to +3, the absolute value of the lowest energy corresponding to the alloy is lower, resulting in a decrease in the absolute value of Δ*E*_M_. This proves that the L1_0_ phases become increasingly stable with the contraction of the optimized volume. However, regardless of any change in volume, the *c*/*a* ratio remains stable: 1.29 for Pd_2_MnGa and 1.3 for Pd_2_FeGa. Furthermore, there is only one minimum located at *c*/*a* > 1 during the tetragonal deformation of Pd_2_MnGa, but two minima for Pd_2_FeGa, with the shallow minimum located at *c*/*a* < 1 and the deeper minimum at *c*/*a* > 1. The stable tetragonal phases of Pd_2_ *YZ* are the states with the lowest energy.

**Figure 10:**
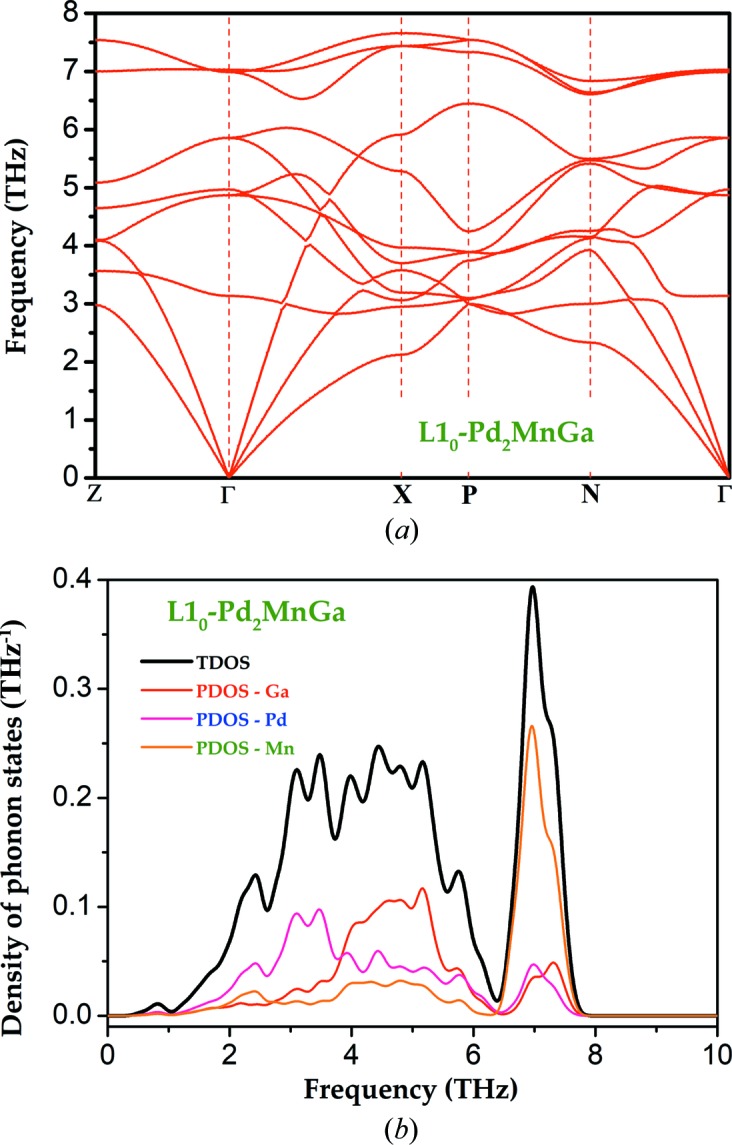
Calculated (*a*) phonon dispersion curves and (*b*) phonon DOS for L1_0_-Pd_2_MnGa.

In order to further validate the stability of our predicted L1_0_ structures, as a special example, we choose Pd_2_MnGa to study its calculated phonon dispersion curves and phonon DOS, as shown in Fig. 10 ▸. It is clear from Fig. 10 ▸(*a*) that L1_0_-Pd_2_MnGa has no imaginary frequencies, indicating the dynamical stability of this material. Furthermore, via analysis of phonon DOS, from which the phonon dispersion originates in Fig. 10 ▸(*b*), we easily found that the three low-frequency (0–4 THz) acoustic phonon curves are mainly attributed to Pd atoms, while the three relatively high-frequency (4–6 THz) optical phonon curves come from Ga atoms and the remaining six high-frequency (6–8 THz) optical phonon curves originate from the Mn atom.

**Figure 11:**
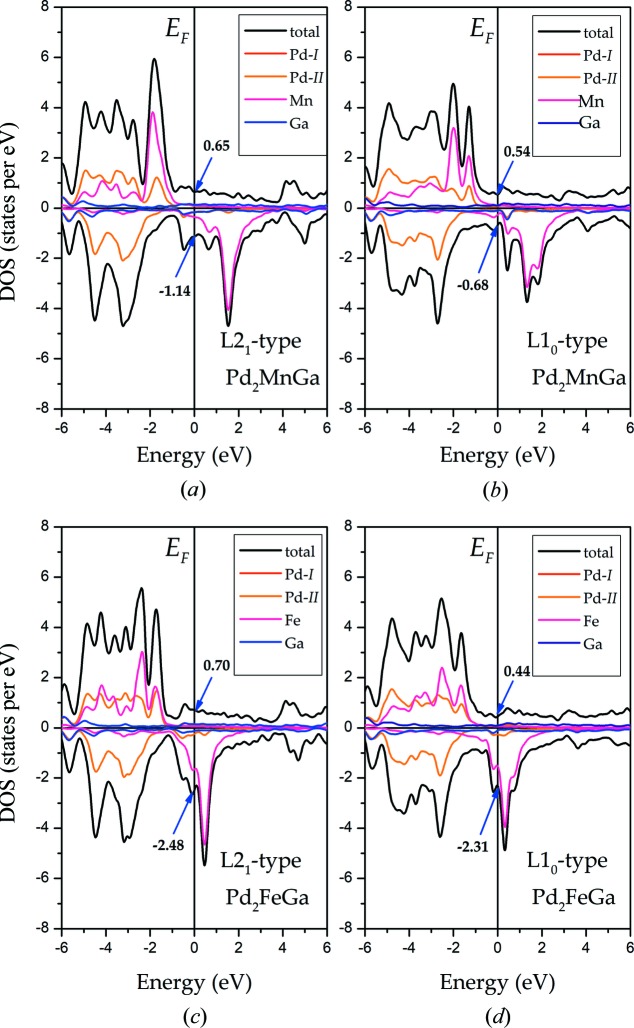
The total and atomic DOS in L2_1_ and the stable tetragonal phases of Pd_2_MnGa and Pd_2_FeGa.

It is clear that whether the cubic L2_1_-type or the tetragonal L1_0_-type structures exhibit metallic properties is explained by the definite value at the *E*_F_ in both majority and minority DOSs. The total DOSs are both almost entirely contributed by the Mn/Fe atoms due to their strong exchange splitting around the Fermi level in these two types. First, we take Pd_2_MnGa as an example: the origin of the tetragonal ground states of these Pd_2_ *YZ* alloys can also be explained based on the DOS structures. It is noted that in the work by Faleev *et al.* (2017 ▸ *b*), one of the contributions to the total energy was the band energy 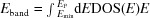, a reduction of the DOS near the *E*_F_ in a tetragonal phase, in conjunction with conservation of the integral for the number of valence electrons 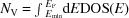, often leads to a lower total energy for the tetragonal phase than for the cubic phase (*E*_min_ here is the minimum energy of the valence bands). As shown in Figs. 11 ▸(*a*) and 11(*b*), in the spin-up channel, the comparatively high total DOS value of 0.65 states per eV at *E*_F_ in the cubic L2_1_ phase becomes a valley DOS structure, having a lower energy of 0.54 states eV^−1^ in the tetragonal L1_0_ phases. Also, a shallow valley turns into a deeper valley in the spin-down channel during the tetragonal deformation, from an absolute value of 1.14 to 0.68 states eV^−1^. Thus, the tetragonal state is more stable than the cubic states for Pd_2_MnGa. We then consider Pd_2_FeGa: a high peak located at *E*_F_ in the minority of the total DOS can be clearly seen in Fig. 11 ▸(*c*). We were excited to find that this high peak with absolute value of 2.48 states per eV shifted to a lower energy, resulting in an apparent valley of only 2.31 states per eV occurring at the *E*_F_ in the tetragonal L1_0_ phase in the minority DOS in Fig. 11 ▸(*d*). Also, the higher DOS moves to a lower energy in the majority of the total DOS, to an extent of 0.26 states per eV, which effectively increases the phase stability of the tetragonal structures. Fermi energy is used as a sensor for the peak-to-valley DOS structure. As a result of the tetragonal deformation, the symmetries of the alloys are destroyed, resulting in a much broader and more shallow DOS structure, or even its disappearance at the *E*_F_; this increases the phase stability.

## 4. Conclusions   

We investigated the atomic ordering competition between XA and L2_1_ types, tetragonal transformation, and phase stability of full-Heusler alloys of Pd_2_ *YZ*. We found that most of these alloys favor crystallization in an L2_1_ structure as opposed to an XA structure, meeting the well known site preference rule. Tetragonal geometric optimization of Pd_2_ *YZ* under the equilibrium cubic type phase indicated that the total energy of the tetragonal L1_0_ phases is lower than that of the cubic L2_1_ phase; thus, a phase transition from cubic to tetragonal is likely to occur in these full-Heusler alloys. We found that the valley-and-peak structure in the vicinity or at the Fermi level in the minority/majority spin channels can be mainly attributed to the tetragonal ground state occurring. Most Pd_2_Co*Z* alloys can overcome the energy barrier between the cubic and tetragonal ground states and possess possible tetragonal transformations as indicated by their large Δ*E*_M_ values, such as the Δ*E*_M_ value of 0.225 eV per f.u. of Pd_2_CoTi which is almost twice that of Mn_2_FeGa. Moreover, the uniform strain can also tune the tetragonal transformation: as the lattice constant increases, Δ*E*_M_ values for Pd_2_ *YZ* decrease. Additionally, these alloys are metallic materials in both cubic and L1_0_ states, and the total magnetic moment mainly originates from the *Y* atoms.

## 5. Outlook   

In this work, we investigated the competition between L2_1_ and L1_0_ structures for 36 palladium-rich Heusler alloys, and we found that 30 of the alloys have a possible phase transition from cubic to tetragonal states, implying that the tetragonal structure is the ground state for these alloys. Moreover, for most of the Pd_2_Co*Z* alloys, the energy difference between the cubic and tetragonal structures is larger than 0.1 per f.u.; that is, only the tetragonal Heusler structure may be observed in these Pd_2_Co*Z* alloys.

To the best of our knowledge, to date there has been little research into the topic of palladium-rich Heusler alloys. Some articles (Winterlik *et al.*, 2008 ▸, 2009 ▸) have reported a few cubic-type Pd_2_-based Heusler alloys, such as Pd_2_ZrAl, Pd_2_HfAl, Pd_2_ZrIn and Pd_2_HfIn, and found that these exhibit excellent superconducting properties. However, based on our current study, much importance should also be attached to the tetragonal-type palladium-rich Heusler alloys, and the experimental preparation of tetragonal-type palladium-rich Heusler alloys is imminent.

Furthermore, Pd_3−*x*_Co_*x*_ *Z*, Pd_3−*x*_Fe_*x*_ *Z* and Pd_3−*x*_Mn_*x*_ *Z* alloys may also be investigated theoretically and experimentally in future work. Because of their tunable crystal structures, Pd_3−*x*_ *Y*_*x*_ *Z* (*Y* = Co, Fe, Mn) alloys can display a wide range of multifunctionalities.

## Supplementary Material

Supporting tables and figures. DOI: 10.1107/S2052252518017578/fc5029sup1.pdf
