# Supplementary material for: Site preference and tetragonal distortion in palladium-rich Heusler alloys
Source: IUCrJ. 2019 Jan 24;6(Pt 2):218–25. doi: 10.1107/S2052252518017578 (PMC6400188; doi:10.1107/S2052252518017578)
Supplement: Supplementary file 1 [file m-06-00218-sup1.pdf]

IUCrJ

Volume 6 (2019)

Supporting information for article:

Site preference and tetragonal distortion in palladium-rich Heusler alloys

Mengxin Wu, Yilin Han, A. Bouhemadou, Zhenxiang Cheng, R. Khenata, Minquan Kuang, Xiangjian Wang, Tie Yang, Hongkuan Yuan and Xiaotian Wang

Table S1. Equilibrium lattice constants and total and atomic magnetic moments of $L2_1$-type Pd_2YZ alloys.

**Alloy Pd_2YZ**	**a** (Å)	**M_t** (μ_B/*f.u.*)	**M_{Pd-1}** (μ_B/*f.u.*)	**M_{Pd-2}** (μ_B/*f.u.*)	**M_Y** (μ_B/*f.u.*)	**M_Z** (μ_B)
Pd_2CoB	5.76	1.7708	0.05	0.05	1.71	-0.04
Pd_2CoAl	6.06	1.78577	0.03	0.03	1.75	-0.03
Pd_2CoGa	6.07	1.81965	0.03	0.03	1.76	-0.03
Pd_2CoIn	6.31	1.89729	0.03	0.03	1.87	-0.03
Pd_2CoTl	6.33	1.91239	0.04	0.04	1.84	-0.03
Pd_2CoSi	5.97	1.64793	0.03	0.03	1.6	-0.02
Pd_2CoGe	6.05	1.73235	0.04	0.04	1.66	-0.01
Pd_2CoSn	6.29	1.79537	0.03	0.03	1.75	-0.02
Pd_2CoPb	6.38	1.86218	0.05	0.05	1.78	-0.02
Pd_2CoP	5.98	1.90466	0.1	0.1	1.7	0
Pd_2CoAs	6.1	1.92489	0.09	0.09	1.75	-0.01
Pd2CoSb	6.29	1.84654	0.06	0.06	1.71	0.01
Pd2FeB	5.82	3.24659	0.15	0.15	2.98	-0.04
Pd2FeAl	6.1	3.23594	0.12	0.12	3.01	-0.01
Pd_2FeGa	6.11	3.06884	0.12	0.12	3.04	-0.01
Pd_2FeIn	6.34	3.32204	0.11	0.11	3.12	-0.01
Pd_2FeTl	6.4	3.35254	0.11	0.11	3.14	-0.01
Pd_2FeSi	6.04	3.19107	0.11	0.11	2.96	0
Pd_2FeGe	6.13	3.29778	0.12	0.12	3.04	0.01
Pd_2FeSn	6.28	3.23755	0.1	0.1	3.04	0.01
Pd_2FePb	6.46	3.38874	0.11	0.11	3.15	0.02
Pd_2FeP	6.07	3.50098	0.19	0.19	3.08	0.04
Pd_2FeAs	6.19	3.52489	0.18	0.18	3.13	0.04
Pd_2FeSb	6.28	3.3978	0.14	0.14	3.05	0.07
Pd_2MnB	5.82	4.02188	0.25	0.25	3.56	-0.05
Pd_2MnAl	6.08<br>6.16 [1]	4.16817	0.23	0.23	3.69	0.02

Pd_2MnGa	6.1	4.17751	0.21	0.21	3.73	0.01
Pd_2MnIn	6.41<br>6.373 [1]	4.32227	0.17	0.17	3.96	0.02
Pd_2MnTl	6.48	4.36747	0.16	0.16	4.03	0.02
Pd_2MnSi	6.02	4.03623	0.18	0.18	3.66	0.01
Pd_2MnGe	6.1	4.09871	0.18	0.18	3.73	0.01
Pd_2MnSn	6.33<br>6.38 [1]	4.16845	0.15	0.15	3.86	0.02
Pd_2MnPb	6.54	4.37833	0.15	0.15	4.05	0.03
Pd_2MnP	6.04	4.18373	0.19	0.19	3.79	0.01
Pd_2MnAs	6.27	4.26885	0.17	0.17	3.94	0
Pd_2MnSb	6.33	4.31233	0.17	0.17	3.91	0.06

References

[1] Webster, P. J., & Tebble, R. S. (1968). Magnetic and chemical order in Pd_2MnAl in relation to order in the Heusler alloys Pd_2MnIn, Pd_2MnSn, and Pd_2MnSb. *Journal of Applied Physics*, 39(2), 471-473.

Table S2. Equilibrium lattice constants and total and atomic magnetic moments of XA-type Pd_2YZ alloys.

**Alloy Pd_2YZ**	**a (Å)**	**M_t (μ_B/*f.u.*)**	**M_{Pd-1} (μ_B/*f.u.*)**	**M_{Pd-2} (μ_B/*f.u.*)**	**M_Y (μ_B/*f.u.*)**	**M_Z (μ_B)**
Pd_2CoB	5.81	1.80618	0.08	0.18	1.61	-0.07
Pd_2CoAl	6.08	1.65988	0.05	0.15	1.52	-0.05
Pd_2CoGa	6.07	1.72374	0.05	0.16	1.556	-0.05
Pd_2CoIn	6.41	1.87099	0.04	0.14	1.74	-0.05
Pd_2CoTl	6.33	1.81253	0.04	0.14	1.66	-0.04
Pd_2CoSi	5.98	1.30642	0.02	0.08	1.25	-0.05
Pd_2CoGe	6.06	1.42584	0.02	0.09	1.35	-0.04
Pd_2CoSn	6.28	1.45054	0.02	0.07	1.4	-0.04
Pd_2CoPb	6.36	1.50025	0.02	0.08	1.44	-0.04
Pd_2CoP	5.99	1.6766	0.05	0.13	1.5	0
Pd_2CoAs	6.1	1.77839	0.06	0.14	1.58	0
Pd_2CoSb	6.27	1.21614	0	0.03	1.21	-0.03
Pd_2FeB	5.8	2.71517	0.04	0.21	2.57	-0.11
Pd_2FeAl	6.1	2.81636	0.2	0.22	2.61	-0.03
Pd_2FeGa	6.11	2.88366	0.03	0.2	2.69	-0.04
Pd_2FeIn	6.32	2.99234	0.03	0.18	2.81	-0.03
Pd_2FeTl	6.38	3.08977	0.04	0.18	2.89	-0.02
Pd_2FeSi	6.03	2.74583	0.06	0.22	2.52	-0.04
Pd_2FeGe	6.05	2.82792	0.06	0.22	2.57	-0.02
Pd_2FeSn	6.27	2.88739	0.05	0.2	2.65	-0.01
Pd_2FePb	6.43	3.0569	0.05	0.2	2.81	-0.01
Pd_2FeP	5.96	2.93078	0.1	0.23	2.6	0
Pd_2FeAs	6.07	2.99786	0.1	0.22	2.67	0
Pd_2FeSb	6.26	2.74105	0.05	0.18	2.53	-0.01
Pd_2MnB	5.94	3.63052	0.06	0.19	3.48	-0.1
Pd_2MnAl	6.2	3.85374	0.05	0.2	3.56	0.04
Pd_2MnGa	6.09	3.7741	0.06	0.2	3.48	0.03

Pd_2MnIn	6.3	3.95839	0.06	0.18	3.66	0.06
Pd_2MnTl	6.36	4.00153	0.04	0.17	3.73	0.04
Pd_2MnSi	6.01	3.31459	0.05	0.16	3.1	0
Pd_2MnGe	6.09	3.52838	0.05	0.15	3.31	0.01
Pd_2MnSn	6.31	3.72078	0.05	0.14	3.49	0.04
Pd_2MnPb	6.53	4.06125	0.06	0.15	3.79	0.06
Pd_2MnP	6	3.4776	0.04	0.18	3.25	0.01
Pd_2MnAs	6.11	3.73324	0.08	0.18	3.44	0.04
Pd_2MnSb	6.31	3.57519	0.02	0.15	3.36	0.04

Table S3. ΔE_M values, c/a ratios and total and atomic magnetic moments of $L1_0$-type Pd_2YZ in their stable phases.

Alloy Pd_2YZ	ΔE_M (eV)	c/a ratio	M_t (μ_B)	M_{Pd} (μ_B)	M_Y (μ_B)	M_Z (μ_B)
Pd_2CoB	0.17155	1.27	1.7708	0.05	1.72	-0.04
Pd_2CoAl	0.15481	1.29	1.91242	0.13	1.69	-0.04
Pd_2CoGa	0.18153	1.3	1.97763	0.14	1.74	-0.04
Pd_2CoIn	0.18286	1.33	2.01185	0.12	1.8	-0.04
Pd_2CoTl	0.22354	1.35	2.0397	0.13	1.81	-0.03
Pd_2CoSi	0.15304	1.3	1.61873	0.06	1.54	-0.03
Pd_2CoGe	0.17714	1.33	1.75641	0.08	1.62	-0.02
Pd_2CoSn	0.16479	1.34	1.71536	0.05	1.65	-0.03
Pd_2CoPb	0.17433	1.37	1.83629	0.07	1.71	-0.02
Pd_2CoP	0.06372	1.31	2.28633	0.24	1.75	0.06
Pd_2CoAs	0.06072	1.34	2.26212	0.22	1.77	0.05
Pd_2CoSb	0.13434	1.36	1.87862	0.12	1.63	0.01
Pd_2FeB	0.09957	1.29	3.30537	0.22	2.95	-0.08
Pd_2FeAl	0.03091	1.26	3.1361	0.14	2.88	-0.03
Pd_2FeGa	0.06703	1.3	3.23349	0.16	2.94	-0.03
Pd_2FeIn	0.04623	1.31	3.2314	0.13	3.01	-0.03
Pd_2FeTl	0.09271	1.34	3.30538	0.14	3.05	-0.03
Pd_2FeSi	0.04330	0.85	3.15002	0.14	2.89	-0.02
Pd_2FeGe	0.06845	0.84	3.24553	0.15	2.97	-0.01
Pd_2FeSn	0.05061	1.27	3.11084	0.09	2.94	-0.01
Pd_2FePb	0.04005	0.83	3.30681	0.12	3.07	-0.01
Pd_2FeP	0.103511	1.38	3.62641	0.3	2.96	0.06
Pd_2FeAs	0.08326	1.4	3.60818	0.28	2.99	0.05
Pd_2FeSb	0.08487	1.38	3.274	0.2	2.83	0.04
Pd_2MnB	0.10918	1.28	4.03993	0.3	3.5	-0.05
Pd_2MnGa	0.04288	1.29	4.13138	0.24	3.64	0.01
Pd_2MnTl	0.02044	1.31	4.35991	0.19	3.94	0.03

Pd_2MnP	0.07809	1.35	4.08563	0.21	3.67	0
Pd_2MnAs	0.01594	1.24	4.11632	0.14	3.87	-0.03
Pd_2MnSb	0.02028	1.25	4.1728	0.16	3.81	0.04

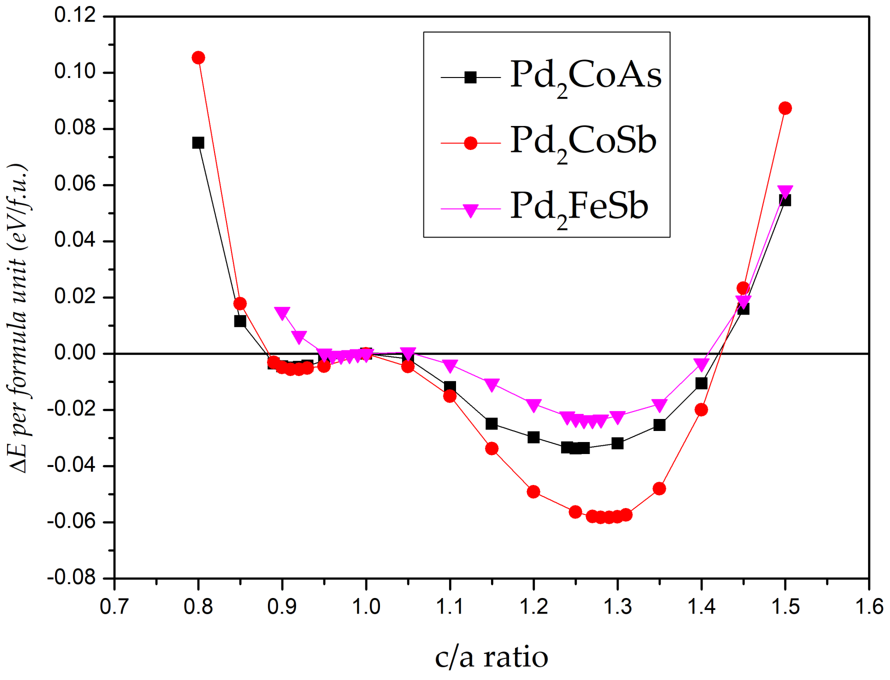

Supplementary Figure S1 Total energies as functions of the c/a ratio for XA-type full-Heusler alloys Pd_2CoSb, Pd_2CoAs, Pd_2FeSb. Through regulating the c/a ratio during the tetragonal deformation, we found that the inverse tetragonal ground state is more stable than the inverse cubic state due to its lower energy.

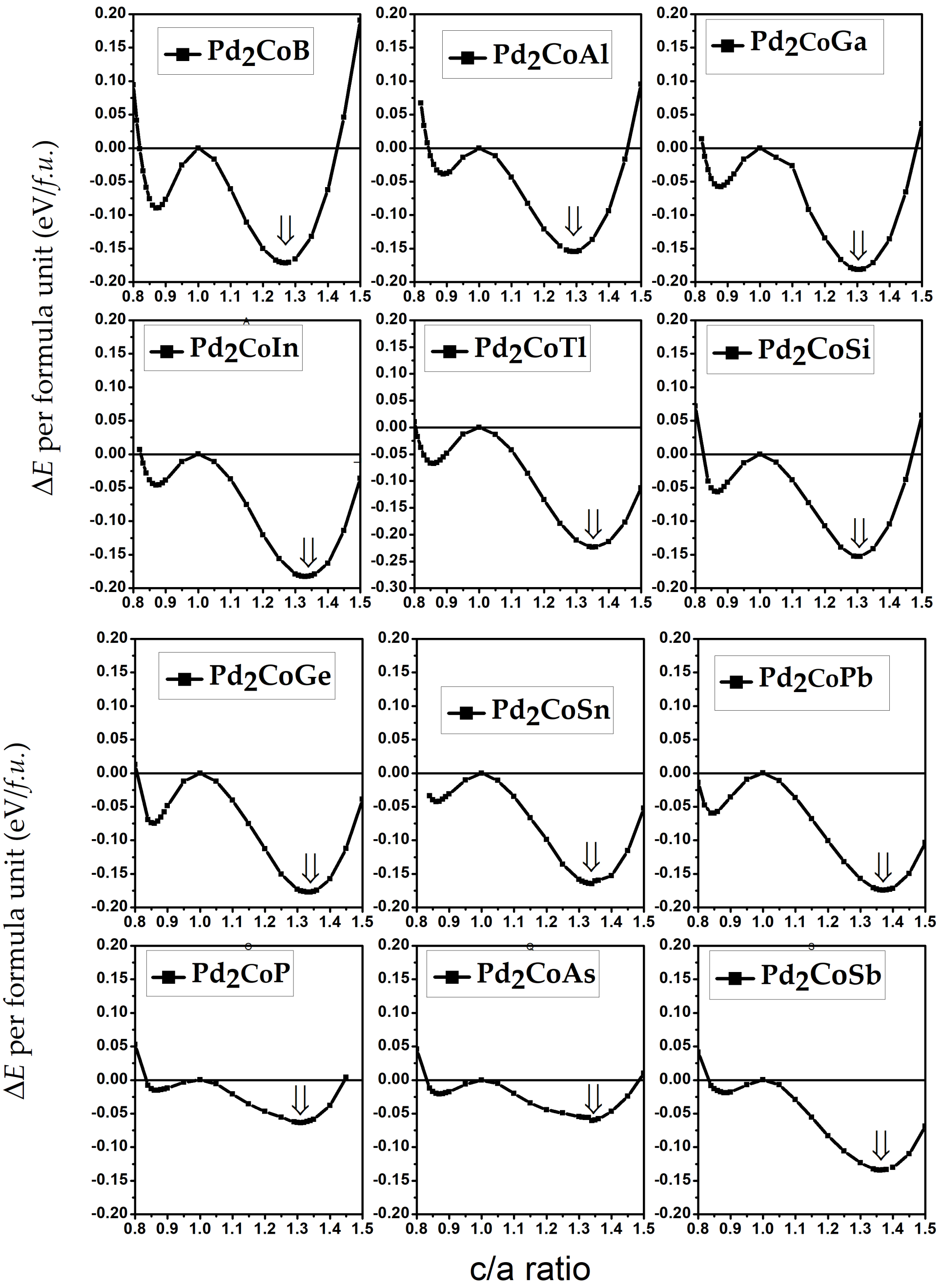

Supplementary Figure S2 Total energies as functions of the c/a ratio for full-Heusler alloys Pd_2CoZ (Z = B, Al, Ga, In, Tl, Si, Ge, Sn, Pb, P, As, Sb) and the corresponding $L1_0$ phases were marked.

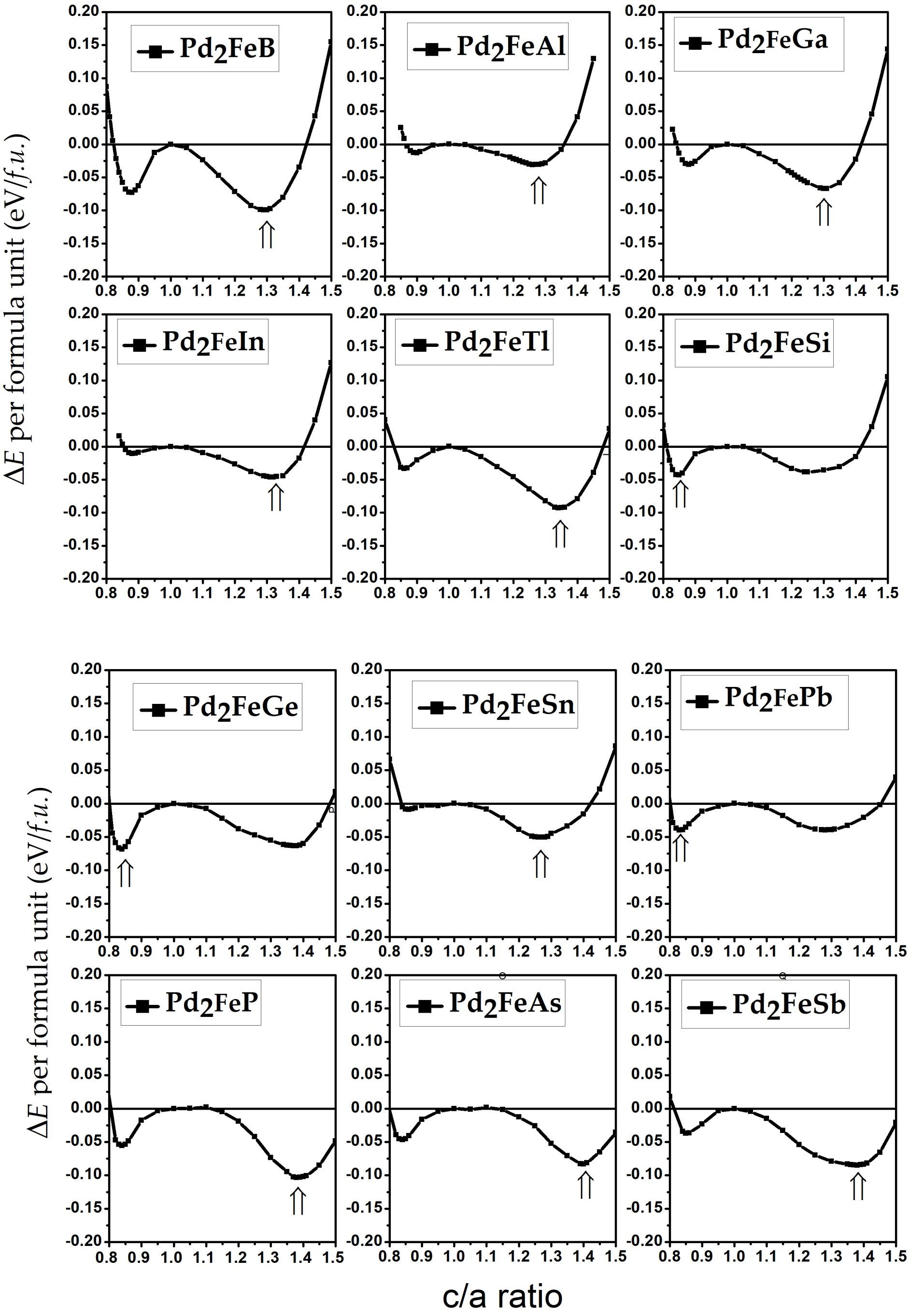

Supplementary Figure S3 Total energies as functions of the c/a ratio for full-Heusler alloys Pd_2FeZ (Z = B, Al, Ga, In, Tl, Si, Ge, Sn, Pb, P, As, Sb) and the corresponding $L1_0$ phases were marked.

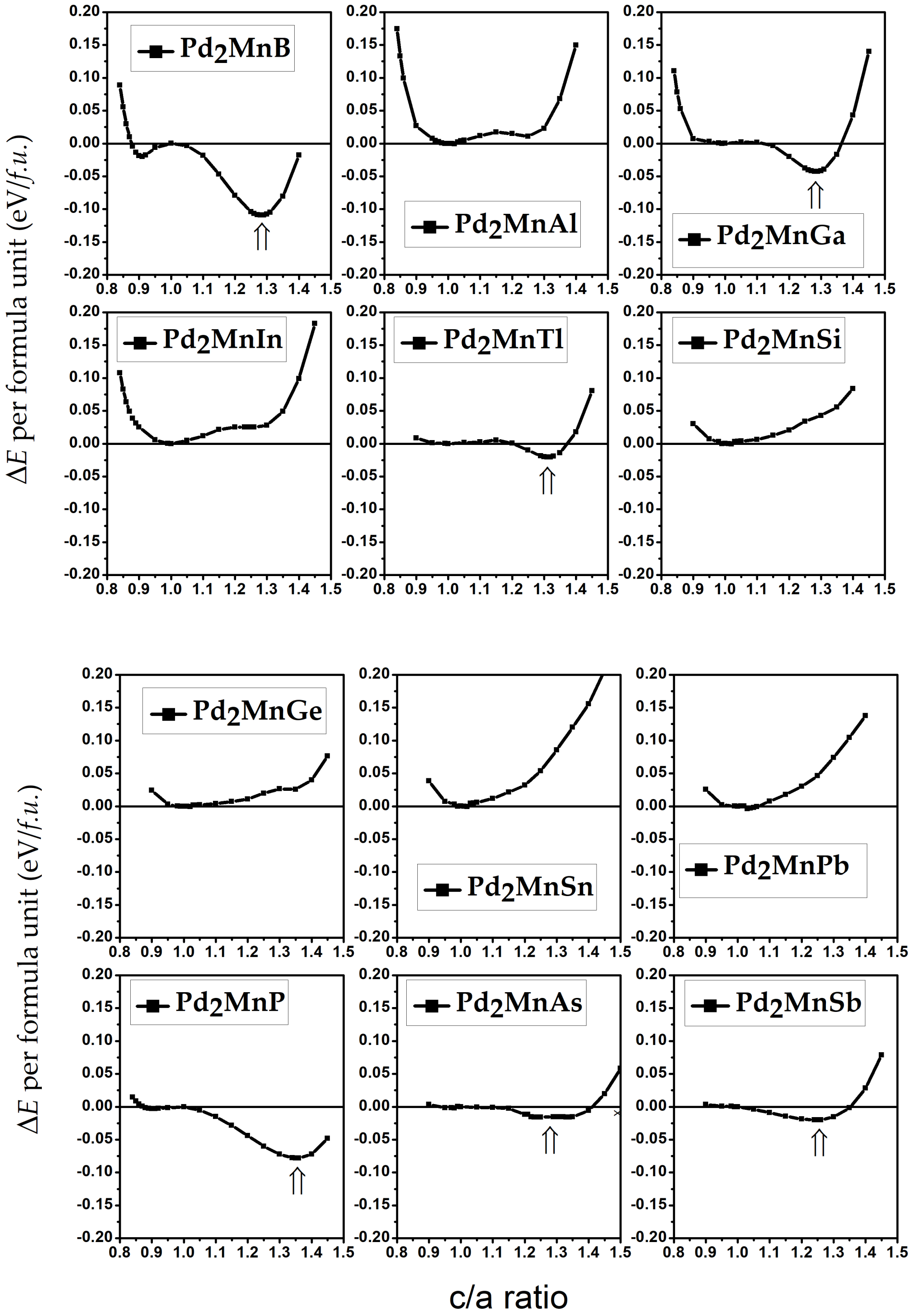

Supplementary Figure S4 Total energies as functions of the c/a ratio for full-Heusler alloys Pd_2MnZ (Z = B, Al, Ga, In, Tl, Si, Ge, Sn, Pb, P, As, Sb) and the corresponding $L1_0$ phases were marked.
